# How to manage sleep in women with ADHD during (peri)menopause?

**DOI:** 10.1192/j.eurpsy.2025.243

**Published:** 2025-08-26

**Authors:** D. Wynchank

**Affiliations:** ISP ADHD bij volwassenen, PsyQ, The Hague, Netherlands

## Abstract

**Abstract:**

For women navigating both ADHD and the (peri)menopausal transition, sleep problems are a common and significant hurdle. This presentation explores effective ways to improve sleep for (peri)menopausal women with ADHD, drawing on research into hormonal, non-hormonal, and behavioural approaches. ADHD can make sleep difficult, contributing to issues like delayed sleep phase, insomnia, and restless legs syndrome/periodic limb movement disorder (RLS/PLMD), with roughly 60% of adults with ADHD screening positive for a sleep disorder. Research indicates that adults with ADHD often take longer to fall asleep and experience more sleep-related challenges than those without ADHD. Similarly, (peri)menopausal women often struggle with sleep disturbances, including poor sleep quality and increased restless legs symptoms. Considering that (peri)menopausal symptoms can also impact cognitive function, addressing these symptoms may lead to better sleep. Menopausal Replacement Therapy (MRT) may be beneficial, especially for women with vasomotor symptoms, as it can improve sleep quality and reduce nighttime awakenings. In addition, multiple studies suggest that bioidentical progesterone can improve sleep quality in perimenopausal women. We will also discuss non-hormonal options and behavioural strategies like cognitive behavioural therapy, exercise, and mindfulness techniques. With a significant percentage of women with ADHD diagnosed with a sleep disorder and prescribed sleep medication, and a similar percentage of perimenopausal women experiencing sleep disturbances, a personalised, integrated approach is key. This includes fine-tuning ADHD medication, managing any co-existing mood issues, and customising treatments to fit individual needs.

**Disclosure of Interest:**

None Declared

